# Neuronal intranuclear inclusion disease is genetically heterogeneous

**DOI:** 10.1002/acn3.51151

**Published:** 2020-08-10

**Authors:** Zhongbo Chen, Wai Yan Yau, Zane Jaunmuktane, Arianna Tucci, Prasanth Sivakumar, Sarah A. Gagliano Taliun, Chris Turner, Stephanie Efthymiou, Kristina Ibáñez, Roisin Sullivan, Farah Bibi, Alkyoni Athanasiou‐Fragkouli, Thomas Bourinaris, David Zhang, Tamas Revesz, Tammaryn Lashley, Michael DeTure, Dennis W. Dickson, Keith A. Josephs, Ellen Gelpi, Gabor G. Kovacs, Glenda Halliday, Dominic B. Rowe, Ian Blair, Pentti J. Tienari, Anu Suomalainen, Nick C. Fox, Nicholas W. Wood, Andrew J. Lees, Matti J. Haltia, Genomics England Research Consortium, John Hardy, Mina Ryten, Jana Vandrovcova, Henry Houlden

**Affiliations:** ^1^ Department of Neurodegenerative Disease Queen Square Institute of Neurology University College London (UCL) London UK; ^2^ Department of Neuromuscular Disease Queen Square Institute of Neurology UCL London UK; ^3^ Queen Square Brain Bank Department of Clinical and Movement Neurosciences Queen Square Institute of Neurology UCL UK; ^4^ Clinical Pharmacology William Harvey Research Institute School of Medicine and Dentistry Queen Mary University of London London UK; ^5^ Center for Statistical Genetics and Department of Biostatistics University of Michigan Ann Arbor Michigan; ^6^ Queen Square Institute of Neurology UCL and the National Hospital for Neurology and Neurosurgery Queen Square London UK; ^7^ University Institute of Biochemistry & Biotechnology PMAS – Arid Agriculture University Rawalpindi Pakistan; ^8^ Department of Neuroscience Mayo Clinic Jacksonville Florida; ^9^ Mayo Clinic Neurodegenerative Research Group Rochester Minnesota; ^10^ Neurological Tissue Bank of the Hospital Clinic‐Institut d’Investigacions Biomediques August Pi I Sunyer (IDIBAPS) Biobank Barcelona Spain; ^11^ Division of Neuropathology and Neurochemistry Department of Neurology Medical University of Vienna Austria; ^12^ University of Toronto Tanz Centre for Research in Neurodegenerative Disease Toronto Canada; ^13^ Neuroscience Research Australia Sydney Australia; ^14^ School of Medical Sciences Faculty of Medicine University of New South Wales Sydney Australia; ^15^ Brain and Mind Centre Sydney Medical School The University of Sydney Sydney Australia; ^16^ Centre for Motor Neuron Disease Research Department of Biomedical Sciences Faculty of Medicine and Health Sciences Macquarie University Sydney NSW Australia; ^17^ Department of Neurology Helsinki University Hospital Helsinki Finland; ^18^ Translational Immunology Research Program Faculty of Medicine University of Helsinki Helsinki Finland; ^19^ Research Programs Unit, Stem Cells and Metabolism University of Helsinki Helsinki 00290 Finland; ^20^ Neuroscience Center HiLife University of Helsinki Helsinki 00290 Finland; ^21^ HUSlab Helsinki University Hospital Helsinki 00290 Finland; ^22^ Dementia Research Centre UCL London Queen Square UK; ^23^ Department of Clinical and Movement Neurosciences Queen Square Institute of Neurology UCL London UK; ^24^ Reta Lila Weston Institute UCL Queen Square Institute of Neurology Wakefield Street London; ^25^ Department of Pathology Faculty of Medicine University of Helsinki Helsinki Finland; ^26^ Genomics England London UK; ^27^ William Harvey Research Institute Queen Mary University of London London EC1M 6BQ UK; ^28^ UK Dementia Research Institute at UCL Queen Square Institute of Neurology UCL London UK; ^29^ NIHR University College London Hospitals Biomedical Research Centre London UK; ^30^ Institute for Advanced Study The Hong Kong University of Science and Technology Hong Kong SAR China

## Abstract

Neuronal intranuclear inclusion disease (NIID) is a clinically heterogeneous neurodegenerative condition characterized by pathological intranuclear eosinophilic inclusions. A CGG repeat expansion in *NOTCH2NLC* was recently identified to be associated with NIID in patients of Japanese descent. We screened pathologically confirmed European NIID, cases of neurodegenerative disease with intranuclear inclusions and applied in silico‐based screening using whole‐genome sequencing data from 20 536 participants in the 100 000 Genomes Project. We identified a single European case harbouring the pathogenic repeat expansion with a distinct haplotype structure. Thus, we propose new diagnostic criteria as European NIID represents a distinct disease entity from East Asian cases.

## Introduction

Neuronal intranuclear inclusion disease (NIID) is a clinically heterogeneous, multi‐system neurodegenerative condition with manifestations comprising cognitive impairment, parkinsonism, and neuropathy at varying ages of onset.^1^ Central to the pathological diagnosis is presence of characteristic intranuclear eosinophilic ubiquitinated inclusions in both neuronal and non‐neuronal cells. Despite the first case being described in 1968,^2^ a CGG repeat expansion in *NOTCH2NLC* has only been found recently to be associated with NIID in Japanese patients.^3^, ^4^ This was prompted by the clinico‐pathological overlap with Fragile X‐associated tremor‐ataxia syndrome (FXTAS)^5^ and increasing recognition of noncoding repeat expansions being crucially causative in neurological disorders.^3^, ^4^ Since these findings, the same expansion has been reported in several East Asian cohorts including Chinese patients with skin‐biopsy proven NIID^6^, ^7^; Chinese essential tremor cases^8^ and Japanese leukodystrophy cases.^9^

Inspired by the high prevalence of this expansion within East Asian patients, we instigated screening for the repeat within Europeans with pathological confirmation of neuronal and/or glial hyaline intranuclear inclusions on brain tissue to further understand the molecular basis of disease. The very similar intranuclear inclusions seen in NIID can occur concomitantly with another proteinopathy. Therefore, we also screened post‐mortem cases with neuronal intranuclear inclusions (NIIs) in other neurodegenerative diseases with the aim of assessing whether clinically heterogeneous presentations converge on a common proteinopathy aggregate. Lastly, we applied *in silico*‐based screening of a deeply‐characterized cohort of 20,536 patients with neurological presentations enrolled in the 100,000 Genomes Project to characterize the prevalence within a predominantly European population.^10^ We show that the *NOTCH2NLC* repeat expansion is a rare cause of NIID in Europeans and that at least two distinct disease entities exist under the name NIID.

## Methods

### Case selection

The study was approved by UCL Institute of Neurology Institutional Review Board. Tissue and DNA samples from other institutions met approval from local ethics boards. Eleven NIID cases (Cases 1–11) were identified from: Queen Square Brain Bank (QSBB)^11^; Spain (IDIBAPS Brain Bank Barcelona)^5^; Finland^12^; Australia (South Australian Brain Bank^13^ and Macquarie University) and USA (Mayo Clinic). Thirteen cases with primary protein misfolding pathology and NIIs (Cases 2‐1 to 2‐13) were included from: QSBB; Austria (Vienna Brain Bank)^14^; IDIBAPS^5^ and Mayo Clinic. Five cases of FTLD‐FUS were also included from IDIBAPS and QSBB (Cases 2‐14 to 2‐18). We used a Japanese patient previously described with *NOTCH2NLC* repeat expansion‐associated NIID (Case J) as a positive control.^3^ DNA extraction from QSBB, Spain, and USA samples of fresh frozen cerebellar tissue was carried out as per Qiagen Gentra Puregene Tissue Kit protocol (concentration ≥ 219.7 ng/µL).

### Repeat‐primed polymerase chain reaction and fragment analysis

**Figure 1:**
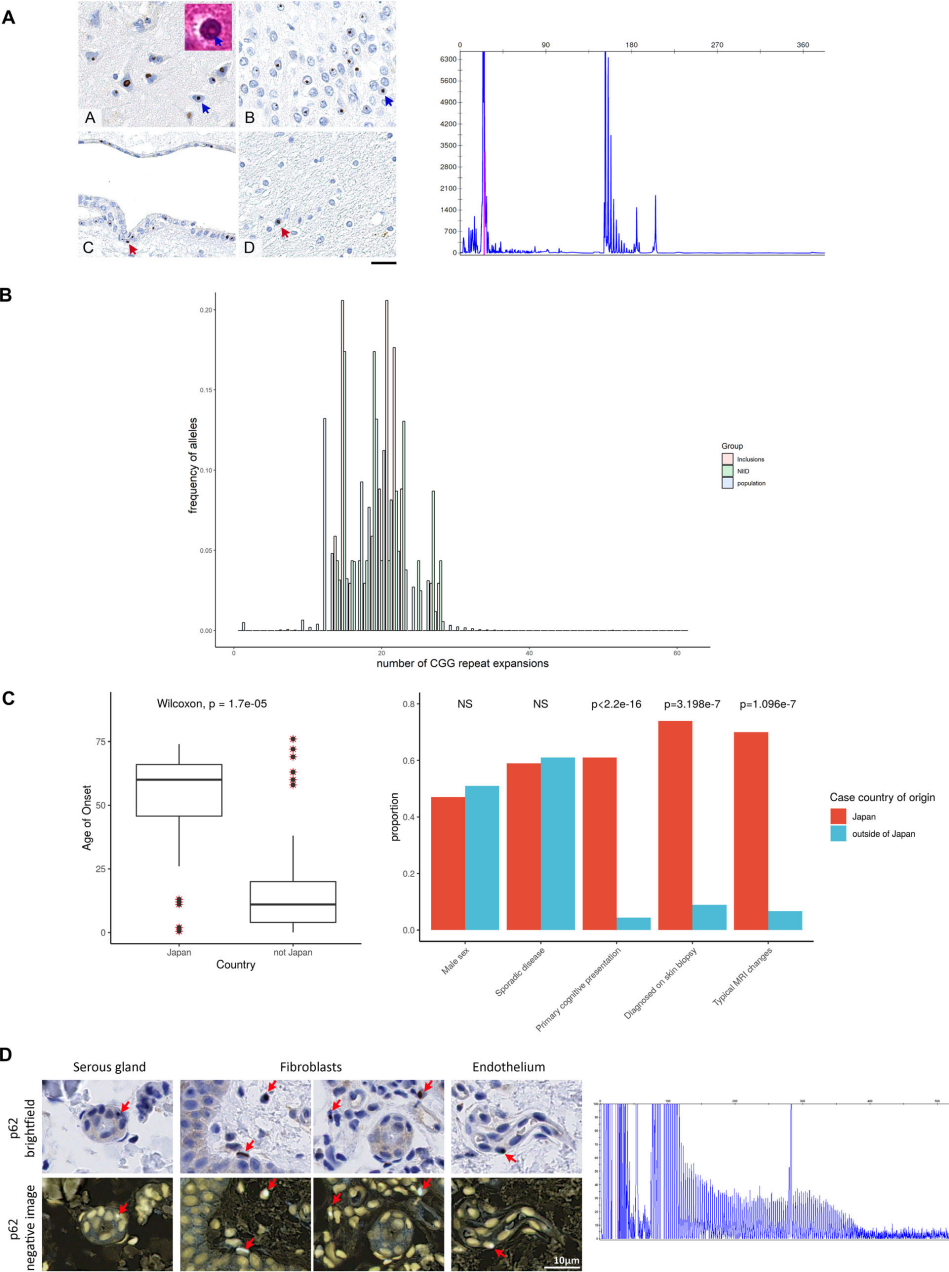
Distribution of *NOTCH2NLC* repeat expansions. Panel A is that of Patient 1^11^: intranuclear p62 immunoreactive inclusions are present in the majority of the neurons across the neocortex (A, blue arrow), dentate gyrus in the hippocampus (B, blue arrow), deep grey nuclei, brainstem nuclei and cerebellar neurons (not shown). The inclusions are eosinophilic on routine haematoxylin and eosin stained sections (inset in A, blue arrow). The intranuclear inclusions are also frequently seen in the ependymal cells (C, red arrow) but only rarely observed in glial cells (D, red arrow). Scale bar: 20 µm in A‐D. The corresponding electropherogram confirms absence of the repeat expansion within this patient. Panel B shows the histogram distributions of the number of CGG repeats in the population (population) (estimated from ExpansionHunter based on 20,536 participants with neurological presentations enrolled into the 100,000 Genomes Project) compared with cases of neuropathologically confirmed NIID within our samples (NIID) and cases with evidence of pathological intranuclear inclusions (inclusions). Panel C summarizes the comparison of clinical characteristics between cases of NIID described within and outside of Japan. Panel D shows brightfield‐positive and brightfield‐negative images for p62 immunoreactivity in the skin biopsy of the patient identified from 100,000 Genomes Project (Case 12). The corresponding electropherogram infers presence of a repeat expansion as seen by the typical sawtooth pattern.

Repeat‐primed polymerase chain reaction (RP‐PCR) was designed as described^3^ to assess for CGG repeat expansion using genomic DNA. RP‐PCR analysis was performed using primers: 5’‐AGCGCCCACAGCAGAGCGGC‐3’; 5’‐CCGGGAGCTGCATGTGTCAGAGGCGGCGGCGGCGGCGG‐3’; 5’‐(FAM)‐CCGGGAGCTGCATGTGTCAGAGG3’, LA taq with GC buffer (TaKaRa Bio) and deaza‐dGTP. The PCR protocol used initial denaturation at 95°C for 5 minutes, followed by 50 cycles of 95°C for 30 seconds, 98°C for 10s, 62°C for 30 seconds and 72°C for 2 minutes. The ramp rate to 95°C and 72°C was 2.5°C per second and that to 62°C was 1.5°C per second^3^. For fragment analysis, 9.2 µL HiDi formamide was combined with 0.5 µL LIZ 500 size standard per 1 µL PCR product. FAM‐labeled PCR products were denatured at 95 °C for three minutes and on ice for three minutes then separated on ABI3730 DNA Analyser (ThermoFisher). Electropherograms were visualized on GENEMAPPER (ThermoFisher). We judged a sawtooth tail pattern in the electropherogram as the disease‐associated repeat expansion (Figure 1). This process was replicated three times, with three positive controls to ensure negative results did not arise from technical error. Estimating repeat size from fragment analysis employed previously described protocol.^3^

### Whole‐genome sequence analysis for repeat expansion

We used ExpansionHunter v.2,^15^, ^16^ a validated tool that identifies repeat expansions using whole‐genome sequencing (WGS) data. We searched for “CGG” repeats within the genomic co‐ordinates of the repeat expansion (Chr1:149390802‐149390841, GRCh38) in a cohort of 20,536 patients with neurological presentation recruited into the 100 000 Genomes Project.^10^ Interruptions within the repeat sequence were accounted for in the algorithm. Ethnicities were estimated using a random forest classifier based on 1,000 Genomes Project as a training dataset.

### Genotyping

Sample processing for Illumina GSAv2.0 arrays was carried out according to Infinium HTS Assay protocol (Illumina Inc.) at UCL Genomics. Three hundred nanogram of DNA was whole‐genome amplified, fragmented, precipitated, and resuspended in hybridization buffer. Samples were hybridized onto Illumina GSA beadchips and incubated at 48 °C for 16 hours. Beadchips were stained then scanned using iScan (Illumina). Total genotyping rate was 0.993. Principal components were calculated using PLINK v.1.9^17^ and population stratification analysis for inferred ancestries using Peddy (Python).

### Haplotype analysis

Haplotype blocks were estimated based on 90% confidence intervals of *D’* disequilibrium statistic for pairs of variants (PLINK^17^). The haplotype analysis was set within the *NOTCH2NL* paralogous region (Chr1:120705588–149410843, GRCh38) containing 380 genotyped SNPs. The genotyped SNP overlap between the three patient groups compared (*NOTCH2NLC* expansion‐negative European NIID, Case 12 and Case J) was high at 96.7% remaining consistent at 96.3% with minor allele frequency (MAF) >0.05.

### Comparisons of clinical characteristics

We reviewed Medline and Pubmed databases for cases of “neuronal intranuclear inclusion disease”; “neuronal intranuclear hyaline disease”; “neuronal intranuclear hyaline inclusion disease” and “intranuclear hyaline inclusion disease,” using key search terms as applied, without a date restriction. We identified 145 independent cases of NIID reported in the literature (April 2019). All statistical analyses were executed in R (version 3.5.1).

## Results

### NIID is genetically and phenotypically heterogeneous

We find no evidence of the repeat to a pathological level within eleven NIID cases of European ancestry confirmed on post‐mortem brain examination (Table 1: Cases 1–11). These cases have been well‐characterized including a monozygotic twin with juvenile‐onset movement disorder, from whom the term NIID was coined^12^; as well as other cases with both juvenile‐onset^11^ and adult‐onset^5^, ^13^ disease. Revisiting the pathology confirmed that NIIs stained positive for p62^11^ further validating the diagnosis (Figure 1A). The median number of CGG repeats in *NOTCH2NLC* was 20 (range 14–28) in these patients (Figure 1B), falling within the range of repeats seen in asymptomatic Far East populations.^3^, ^4^ This suggests genetically heterogeneous mechanisms underlie NIID in European patients. In support of this diverse underlying molecular mechanism is the dichotomy in clinical presentation between non‐Japanese and Japanese NIID cases. Of 145 reported NIID cases, two thirds are from Japan (100 cases) and are of an older age of onset compared to non‐Japanese cases (median (IQR): 60 years (46–66) and 11 years (4–20) respectively, Wilcoxon rank sum *P*‐value = 1.67e^‐5^). Most Japanese patients had a primary cognitive presentation (61%), with a large proportion of cases having pathognomonic MRI changes at the corticomedullary junction (70%). Furthermore, 74% of Japanese cases were diagnosed on antemortem skin biopsy compared with ~ 9% of non‐Japanese cases reflecting the lack of extraneuronal involvement in cases outside of Japan^12^ (Figure 1C). Deeper comparison of the inclusions has demonstrated differences also in their composition; inclusions were likely filamentous in European cases^12^ without the fine granular material reported in Japanese cases.^1^

**Table 1:** Estimated number of repeat expansions in cases with pathologically confirmed NIID and cases with evidence of neuronal intranuclear inclusions on pathological examination of the brain.

	Case ID	Estimated number of CGG repeats		Age of onset	Sex	Family history	Country of origin	Clinical Diagnosis/ Presentation pre‐biopsy	Main pathological findings and diagnosis	Other pathological findings
		Allele 1	Allele 2							
Pathologically‐confirmed NIID	1^1^	21	‐	17	M	Yes	UK	Parkinsonism, tremor, bulbar and autonomic symptoms. Died aged 24 years.	NIID: widespread neuronal hyaline intranuclear inclusions immunoreactive for ubiquitin and p62	See Figure 1C
	2^2^	22	28	33	M	Yes	Australia	Slowly progressive motor and sensory neuronopathy with ataxia. Death at 46 years.	NIID: eosinophilic neuronal intranuclear inclusions	Degeneration of substantia nigra, medial thalamus and cerebellum
	3^2^	15	20	60s	F	No	Australia	Unknown presentation. Death aged 67 years.	NIID: cortical neurons especially large pyramidal cells show eosinophilic intranuclear inclusions	No overt neuronal loss from the cerebral cortex and no reactive astrogliosis
	4	15	23	52	F	No	Australia	Slowly progressive primary lateral sclerosis. Death aged 72 years.	NIID: neuronal and astrocytic intranuclear inclusions throughout the cerebral cortex	Upper motor neuron loss and lateral corticospinal tract degeneration
	5^3^	19	22	11	F	Yes (MZ twin)	Finland	Ataxia, rage, seizures and extrapyramidal symptoms. Death aged 21years.	NIID: inclusion bodies in most nerve cell types of central and peripheral nervous systems	Inclusions also seen in the retina and subtotal loss of nigral neurons
	6^4^	15	25	49	F	Yes	Spain	Ataxia. Death aged 62 years.	NIID: abundant glial nuclear inclusions	Rosai‐Dorfman disease (Case 3 Gelpi *et al.*)
	7^4^	16	23	82	F	Yes	Spain	Dementia. Death aged 84 years.	NIID: abundant glial nuclear inclusions	ARTAG and SVD (Case 2 Gelpi *et al.)*
	8	17	23	26	F	No	USA	Clinical diagnosis unclear	NIID	
	9	15	19	84	M	No	USA	Alzheimer’s disease, ataxia	NIID: intranuclear hyaline inclusions in neurons and glia in widespread areas of the brain	Hippocampal sclerosis, argyrophilic grain disease, Braak 0, Thal 1, TDP 1
	10	14	27	69	M	No	USA	Diagnosed clinically with NIID	NIID: neuronal intranuclear inclusions	
	11	19	‐	80	M	No	USA	Unknown presentation	NIID	Inferior olivary hypertrophy
	12	19	expanded	51	F	No	Ukraine	Relapsing encephalopathy and migraines	Antemortem skin biopsy contains p62 positive intranuclear inclusions	

	Case ID	Estimated number of CGG repeats		Age of onset	Sex	Family history	Country of origin	Clinical Diagnosis/ Presentation pre‐biopsy	Main pathology	Presence of p62/ ubiquitin‐positive and TDP43 or FUS‐negative NIIs
		Allele 1	Allele 2							
Other pathology with neuronal intranuclear inclusions (NIIs)	2‐1	15	19	49	F	Unknown	UK	Parkinson’s disease	Parkinson’s disease, Braak 6 (diffuse neocortical)	Medial temporal lobe
	2‐2	15	23	49	M	Yes (PD in distant family)	UK	Progressive supranuclear palsy	Parkinson’s disease, Braak 4 (limbic)	Medial temporal lobe
	2‐3	15	22	51	F	No	UK	Corticobasal syndrome	Alzheimer’s disease with amygdala‐restricted Lewy pathology	Medial temporal lobe
	2‐4	15	21	56	M	Unknown	UK	Progressive supranuclear palsy	Progressive supranuclear palsy	Medial temporal lobe and neocortex
	2‐5	20	‐	78	F	Unknown	UK	Corticobasal syndrome	Corticobasal degeneration	Medial temporal lobe, neocortex and cerebellum
	2‐6	14	22	60	M	No	UK	Atypical progressive aphasia	Intermediate level Alzheimer’s disease pathological change	Medial temporal lobe
	2‐7	15	22	83	M	No	UK	Parkinson’s disease	Parkinson’s disease, Braak 5 (limbic)	Medial temporal lobe
	2‐8	18	27	64	M	No	UK	Corticobasal syndrome	Lewy pathology, Braak 5 (limbic); Cerebellar degeneration of unknown nature	Medial temporal lobe, neocortex and cerebellum
	2‐9	18	27	66	F	No	USA	Dementia	Dementia with Lewy bodies	Present
	2‐10^4^	22	23	47	M	Yes	Spain	Rapidly progressive ataxia/ parkinsonism	Fatal familiaI insomnia and incidental inclusions (Case 4 in Gelpi *et al.* 2017)	Hippocampus
	2‐11	21	22	77	M	Unknown	Spain	FXTAS	FXTAS, NFT II, AGD I and SVD	Present
	2‐12	21	28	86	N	Unknown	Austria	Severe dementia	Vascular encephalopathy, AGD, ARTAG, AD A2B2C2, CAA type 1 + 2	Present
	2‐13^5^	15	20	63	F	Unknown	Austria	Rapidly progressive dementia, pyramidal and extrapyramidal symptoms	MM1/MV1 CJD, Braak and Braak stage II	Cerebellar Purkinje cells
	2‐14	21	‐	56	M	Unknown	Spain	Fronto‐temporal dementia	FTLD‐ALS‐FUS (ubiquitin), LBD (olfactory bulb)	None
	2‐15	19	23	69	F	No	UK	Fronto‐temporal dementia	FTLD‐FUS	None (NIFID)
	2‐16	16	21	44	M	Unknown	UK	Fronto‐temporal dementia	FTLD‐FUS	None (NIFID)
	2‐17	20	21	41	F	No	UK	Fronto‐temporal dementia	FTLD‐FUS	None (NIFID)
	2‐18	14	22	49	M	No	UK	Fronto‐temporal dementia	FTLD‐FUS	None (FTLD‐U)

Estimated number of CGG repeats using fragment analysis in our patients with NIID (Cases 1 to 12) and in other cases with concomitant intranuclear inclusions and with inclusions associated to other proteinopathies (Cases 2‐1 to 2‐13 and cases of FTLD‐FUS: Cases 2‐14 to 2‐18). Where the sizing is not applicable (‐), it is likely that the allele may be homozygous for the number of repeats in that patient providing overlapping traces and this allele is not expanded as no sawtooth pattern is visualized in comparison to our positive control. ABC score: A, amyloid phase according to Thal; B, Braak and Braak neurofibrillary stage; C, neuritic plaque score according to CERAD (each score ranges from 0 to 3); AD, Alzheimer's disease neuropathological changes; AGD, argyrophilic grain disease; ARTAG, aging‐related tau astrogliopathy; CAA, cerebral amyloid angiopathy; CJD, Creutzfeldt‐Jakob disease; FTLD, frontotemporal dementia; FTLD‐FUS, FTLD‐fused in sarcoma subtype; FTLD‐ALS‐FUS, FTLD and amyotrophic lateral sclerosis of FUS‐subtype; FXTAS, fragile X‐associated tremor/ataxia syndrome; LBD, Lewy body disease; MM, methionine homozygosity at codon 129 of the *PRNP* gene; MV, methionine valine heterozygous genotype at codon 129 of the *PRNP* gene; MZ twin, monozygotic twin; NIFID, neuronal intermediate filament inclusion disease; NIID, neuronal intraneuronal inclusion disease; NIIs, neuronal intranuclear inclusions; NFT, neurofibrillary tangles; PD, Parkinson’s disease; SVD, small vessel disease.

### *NOTCH2NLC* repeat expansion does not underlie other neurodegenerative diseases with secondary intranuclear inclusions

Further confounding the diagnostic definition of NIID is the presence of similar intranuclear inclusions with concomitant protein‐misfolding pathology.^5^ FXTAS was excluded from these cases. To investigate the underlying pathophysiology of such disorders, we screened a cohort of 13 cases with primary pathology in addition to NIIs (Table 1: Cases 2‐1 to 2‐13, Supplementary Figure S1). Within QSBB, ten cases were found to have intranuclear inclusions with positive staining for p62 and ubiquitin out of 850 brain samples. The other cases have been previously reported^5^, ^14^ in a range of presentations such as with coexisting prion disease.^14^ We further screened specific cases of FTLD‐FUS subtype (Table 1: Cases 2‐14 to 2‐18) where the alike intranuclear inclusions have FUS recruited within. We also found no evidence of the repeat expansion within this cohort, which harbour similar estimated CGG repeats as seen in the asymptomatic population (median 20.5, IQR 16–22) (Figure 1B). This suggests that the abnormal repeat expansion in *NOTCH2NLC* is not the only driver for diseases with NIIs and highlights that multiple pathways are likely to converge on the end‐product of intranuclear inclusion formation.

### Frequency of repeat expansion within the European population

We have shown that the repeat expansion is not found in any of our European patients with pathologically‐proven NIID compared to pathogenic expansions in 93–100% of Japanese and Chinese patients.^3^, ^4^ Leveraging the availability of WGS data in a large cohort of 20 536 deeply phenotyped participants presenting with neurological symptoms recruited in the 100 000 Genomes Project,^10^ we found the median number of *NOTCH2NLC* CGG repeats to be 20 (IQR 16–22) within this population (Figure 1B). The number of repeat expansions in our cohort of NIID patients and in those with pathological intranuclear inclusions did not differ significantly from this ‘background’ population (ANOVA *p> *0.05). Furthermore, there were no significant differences in the number of repeats among ethnic groups (Supplementary Figure S2). Fragment analysis was used to verify the expansion size in ten individuals who had an estimated repeat size greater than 40 on one allele as ascertained using ExpansionHunter. In a patient with 58 repeats on one allele estimated from ExpansionHunter, fragment analysis demonstrated a pathogenic repeat expansion in a 59‐year‐old woman of Ukrainian ancestry who presented with a 10‐year history of recurrent encephalopathy and migraines (Case 12). The patient was reviewed with respect to these results and subsequent skin biopsy revealed intranuclear p62 and ubiquitin‐positive inclusions, confirming a diagnosis of NIID (Figure 1D).

Prompted by our observation of the low prevalence (approximately 1 in 20,000) of the pathogenic repeat expansion within a European population and lack of expansion within pathologically‐confirmed cases, analyses of principal components and inferred genetic ancestry showed that the Ukrainian patient (Case 12) had no overlapping ancestry with the Japanese patient (Case J) (Supplementary Figure S3). Analysis of the entire *NOTCH2* region encompassing associated paralogs revealed 27 haplotype blocks from the genotyped SNPs although no SNPs overlapped with expansion‐containing region. This showed differing haplotypes for the Ukrainian patient (Case 12), European *NOTCH2NLC*‐CGG‐negative NIID patients (Cases 1–11) and the expansion‐positive Japanese patient (Case J), even for SNPs with MAF>0.05 (Supplementary Table S1). Thus, presence of the rare repeat expansion in our European patient has likely arisen from a separate founder effect to that seen in Japanese cases.

## Discussion

**Figure 2:**
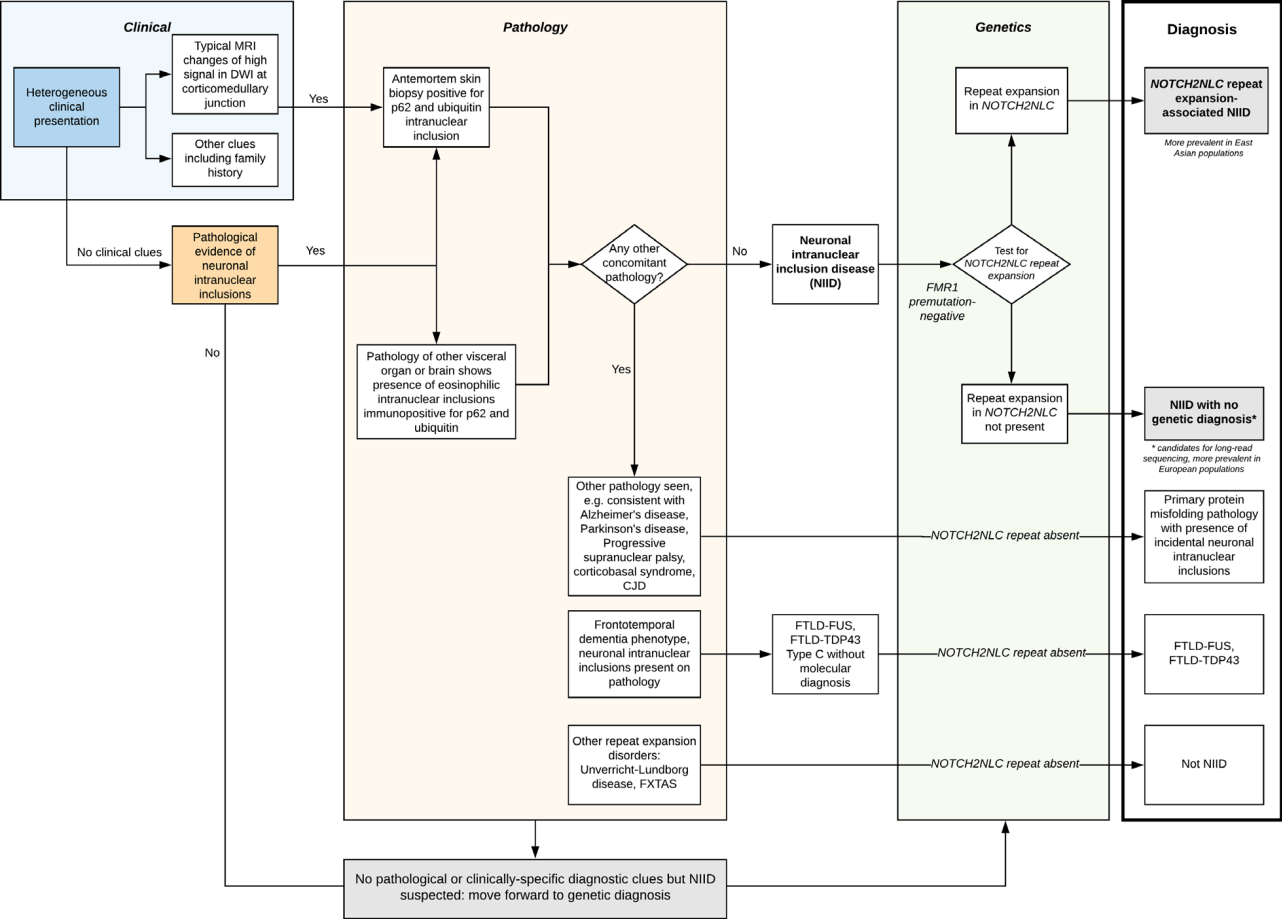
Proposed diagnostic criteria for neuronal intranuclear inclusion‐related diseases. The classification is based on clinical, pathological and genetic criteria. MRI: Magnetic resonance imaging. DWI: diffusion‐weighted imaging. CJD: Creutzfeldt‐Jakob disease. FXTAS: Fragile X‐associated tremor/ataxia syndrome. FTLD‐FUS: frontotemporal dementia‐fused in sarcoma subtype. FTLD‐TDP43: frontotemporal dementia with transactive response DNA binding protein 43 kDa‐positive inclusions.

These results suggest that European NIID cases arise through a separate pathophysiological process to East Asian patients despite both diseases converging on the same signature of abnormal intranuclear inclusions. These differences in genetic, clinical, and pathological features suggest that at least two distinct disease entities exist under the name NIID. While Far East cases are driven by repeat expansion in *NOTCH2NLC*, in the single patient of European ancestry diagnosed with NIID due to *NOTCH2NL*C repeat expansion, haplotype analysis suggested a separate, rarer, founder mutation than that in Japanese cases. Further characterization of the genetic associations with NIID in other populations would be important although we are limited in the number of cases available. We therefore propose new criteria for characterization of NII‐associated disorders (Figure 2) distinguishing between diseases with primary and secondary NIIs partitioned by pathological and molecular features. Thus, our findings are important by showing that the *NOTCH2NLC* repeat expansion is not the only cause underlying NIID pathogenesis or NII formation.

## Genomics England Research Consortium

Ambrose J. C.^1^, Arumugam P.^1^, Baple E. L.^1^, Bleda M.^1^, Boardman‐Pretty F.^1,2^, Boissiere J. M.^1^, Boustred C. R.^1^, Brittain H.^1^, Caulfield M. J.^1,2^, Chan G. C.^1^, Craig C. E. H.^1^, Daugherty L. C.^1^, de Burca A.^1^, Devereau A.^1^, Elgar G.^1,2^, Foulger R. E.^1^, Fowler T.^1^, Furió‐Tarí P.^1^, Hackett J. M.^1^, Halai D.^1^, Hamblin A.^1^, Henderson S.^1,2^, Holman J. E.^1^, Hubbard T. J. P.^1^, Ibáñez K.^1,2^, Jackson R.^1^, Jones L. J.^1,2^, Kasperaviciute D.^1,2^, Kayikci M.^1^, Lahnstein L.^1^, Lawson K.^1^, Leigh S. E. A.^1^, Leong I. U. S.^1^, Lopez F. J.^1^, Maleady‐Crowe F.1, Mason J.^1^, McDonagh E. M.^1,2^, Moutsianas L.^1,2^, Mueller M.^1,2^, Murugaesu N.^1^, Need A. C.^1,2^, Odhams C. A.^1^, Patch C.^1,2^, Perez‐Gil D.^1^, Polychronopoulos D.^1^, Pullinger J.^1^, Rahim T.^1^, Rendon A.^1^, Riesgo‐Ferreiro P.^1^, Rogers T.^1^, Ryten M.^1^, Savage K.^1^, Sawant K.^1^, Scott R. H.^1^, Siddiq A.^1^, Sieghart A.^1^, Smedley D.^1,2^, Smith K. R.^1,2^, Sosinsky A.^1,2^, Spooner W.^1^, Stevens H. E.^1^, Stuckey A.^1^, Sultana R.^1^, Thomas E. R. A.^1,2^, Thompson S. R.^1^, Tregidgo C.^1^, Tucci A.^1,2^, Walsh E.^1^, Watter S. A.^1^, Welland M. J.^1^, Williams E.^1^, Witkowska K.^1,2^, Wood S. M.^1,2^, Zarowiecki M.^1^.

^1^Genomics England, London, UK

^2^William Harvey Research Institute, Queen Mary University of London, London, EC1M 6BQ, UK.

## Author Contributions

ZC, WYY, and HH designed the study. ZC, WYY, SE, RS, FB, AF, and TB performed experimental analyses for the study. ZJ provided pathological interpretation and analysis of samples from QSBB. ZC, AT, PS, SAGT, KIG, DZ, JV, and MR carried out either the haplotype analyses, analyses of Genomics England data, provided by GERC and other data analyses. JH, TR, TL, MD, DWD, KAJ, EG, GGK, GH, DBR, IB PT, ASW, NCF, NWW, AJL, and MJH all provided pathological samples, or patient data. HH, ZC, WYY, and JV conceived and designed the study. HH, JV, and MR supervised the project. All authors discussed the results and contributed to the final manuscript.

## Conflict of Interest

The authors declare no competing interests.

## Supporting information


**Figure S1.** Cases with neuronal intranuclear inclusions and FTLD‐FUS. A and A1 show brightfield‐positive and brightfield‐negative p62 immunoreactive intranuclear inclusions in pyramidal neurones of hippocampus (blue arrows highlight some of the inclusions, Case 2‐5). B and B1 show brightfield‐positive and brightfield‐negative p62 immunoreactive intranuclear inclusions in the inferior temporal gyrus in FTLD‐FUS (red arrows, Case 2‐18). Scale bar: 20µm in A and A1, 10 µm in B and B1.
**Figure S2.** Distribution of repeat expansion sizes across different ethnic groups within 100,000 Genomes Project. The size of repeat expansions shown here are estimated using ExpansionHunter with ethnicities estimated from WGS data using random forest classifier trained on 1,000 Genomes Project data. Abbreviations for populations are as follows: European (EUR); East Asian (EAS); American (AMR); South Asian (ASI); African (AFR).
**Figure S3.** Principal component analysis stratified by self‐reported ethnicity (A) and inferred ancestry compared to 1000 Genomes Project (1kg) (B). Panel A shows the representative principal component analysis across three principal components (PCs) compared between European NIID cases (Cases 1–11: pathologically confirmed cases with negative *NOTCH2NLC* repeat expansion); Case 12 (Ukrainian patient with positive *NOTCH2NLC* repeat expansion); Case J (Japanese patient with known repeat expansion) genotyped on the same GSA chip in the same run. Principal components were calculated using PLINK v.1.9 and shows clustering of Case 12 with other European NIID cases. In Panel B, the solid dots indicate the ancestries from the 1000 Genomes Project while the circles indicate inferred ancestries based on population stratification analysis for our genotyped samples: Cases 1–12 and case J were grouped (across three PCs) as expected to their respective inferred ancestries as estimated from 1000 Genomes Project. Abbreviations for populations are as follows: European (EUR); East Asian (EAS); American (AMR); South Asian (SAS); African (AFR).
**Supplementary Table S1.** Haplotype blocks within the *NOTCH2NL* region of interest. Alleles at sites of SNPs on chromosome 1 (GRCh38) within the *NOTCH2NL* paralogous region of interest, with REF (reference) and ALT (alternate) SNPs at those positions. SNPs denoted by * indicate a MAF>0.05. Haplotype blocks are estimated using PLINK as described. Haplotypes differ between cases of European ancestry (Cases 1–11) compared with Case J (Japanese patient with known repeat expansion) and Case 12 (patient identified from the 100 000 Genomes Project to have the repeat expansion). Comparison is also made with cases with evidence of pathological neuronal intranuclear inclusions.
