# Supplementary material for: Neuronal intranuclear inclusion disease is genetically heterogeneous
Source: Ann Clin Transl Neurol. 2020 Aug 10;7(9):1716–25. doi: 10.1002/acn3.51151 (PMC7480908; doi:10.1002/acn3.51151)
Supplement: Supplementary file 1 — Figure S1. Cases with neuronal intranuclear inclusions and FTLD‐FUS. A and A1 show brightfield‐positive and brightfield‐negative p62 immunoreactive intranuclear inclusions in pyramidal neurones of hippocampus (blue arrows highlight some of the inclusions, Case 2‐5). B and B1 show brightfield‐positive and brightfield‐negative p62 immunoreactive intranuclear inclusions in the inferior temporal gyrus in FTLD‐FUS (red arrows, Case 2‐18). Scale bar: 20µm in A and A1, 10 µm in B and B1. Figure S2. Distribution of repeat expansion sizes across different ethnic groups within 100,000 Genomes Project. The size of repeat expansions shown here are estimated using ExpansionHunter with ethnicities estimated from WGS data using random forest classifier trained on 1,000 Genomes Project data. Abbreviations for populations are as follows: European (EUR); East Asian (EAS); American (AMR); South Asian (ASI); African (AFR). Figure S3. Principal component analysis stratified by self‐reported ethnicity (A) and inferred ancestry compared to 1000 Genomes Project (1kg) (B). Panel A shows the representative principal component analysis across three principal components (PCs) compared between European NIID cases (Cases 1–11: pathologically confirmed cases with negative NOTCH2NLC repeat expansion); Case 12 (Ukrainian patient with positive NOTCH2NLC repeat expansion); Case J (Japanese patient with known repeat expansion) genotyped on the same GSA chip in the same run. Principal components were calculated using PLINK v.1.9 and shows clustering of Case 12 with other European NIID cases. In Panel B, the solid dots indicate the ancestries from the 1000 Genomes Project while the circles indicate inferred ancestries based on population stratification analysis for our genotyped samples: Cases 1–12 and case J were grouped (across three PCs) as expected to their respective inferred ancestries as estimated from 1000 Genomes Project. Abbreviations for populations are as follows: European (EUR); East Asian [file ACN3-7-1716-s001.docx]

***Supplementary Data***

**Neuronal intranuclear inclusion disease is genetically heterogeneous**

**Legends**

**Supplementary Figure 1. Cases with neuronal intranuclear inclusions and FTLD-FUS.** A and A1 show brightfield-positive and brightfield-negative p62 immunoreactive intranuclear inclusions in pyramidal neurones of hippocampus (blue arrows highlight some of the inclusions, **Case 2-5**). B and B1 show brightfield-positive and brightfield-negative p62 immunoreactive intranuclear inclusions in the inferior temporal gyrus in FTLD-FUS (red arrows, **Case 2-18**). Scale bar: 20µm in A and A1, 10 µm in B and B1.

**Supplementary Figure 2. Distribution of repeat expansion sizes across different ethnic groups within 100,000 Genomes Project.** The size of repeat expansions shown here are estimated using ExpansionHunter with ethnicities estimated from WGS data using random forest classifier trained on 1,000 Genomes Project data. Abbreviations for populations are as follows: European (EUR); East Asian (EAS); American (AMR); South Asian (ASI); African (AFR).

**Supplementary Figure 3. Principal component analysis stratified by self-reported ethnicity (A) and inferred ancestry compared to 1000 Genomes Project (1kg) (B).** Panel A shows the representative principal component analysis across three principal components (PCs) compared between European NIID cases (Cases 1 – 11: pathologically-confirmed cases with negative *NOTCH2NLC* repeat expansion); Case 12 (Ukrainian patient with positive *NOTCH2NLC* repeat expansion); Case J (Japanese patient with known repeat expansion) genotyped on the same GSA chip in the same run. Principal components were calculated using PLINK v.1.9 and shows clustering of Case 12 with other European NIID cases. In Panel B, the solid dots indicate the ancestries from the 1,000 Genomes Project while the circles indicate inferred ancestries based on population stratification analysis for our genotyped samples: Cases 1 – 12 and case J were grouped (across three PCs) as expected to their respective inferred ancestries as estimated from 1,000 Genomes Project. Abbreviations for populations are as follows: European (EUR); East Asian (EAS); American (AMR); South Asian (SAS); African (AFR).

**Supplementary Table 1. Haplotype blocks within the *NOTCH2NL* region of interest.**

Alleles at sites of SNPs on chromosome 1 (GRCh38) within the *NOTCH2NL* paralogous region of interest, with REF (reference) and ALT (alternate) SNPs at those positions. SNPs denoted by * indicate a MAF > 0.05. Haplotype blocks are estimated using PLINK as described. Haplotypes differ between cases of European ancestry (Cases 1 – 11) compared with Case J (Japanese patient with known repeat expansion) and Case 12 (patient identified from the 100,000 Genomes Project to have the repeat expansion). Comparison is also made with cases with evidence of pathological neuronal intranuclear inclusions.

**Supplementary Figures and Tables**

**
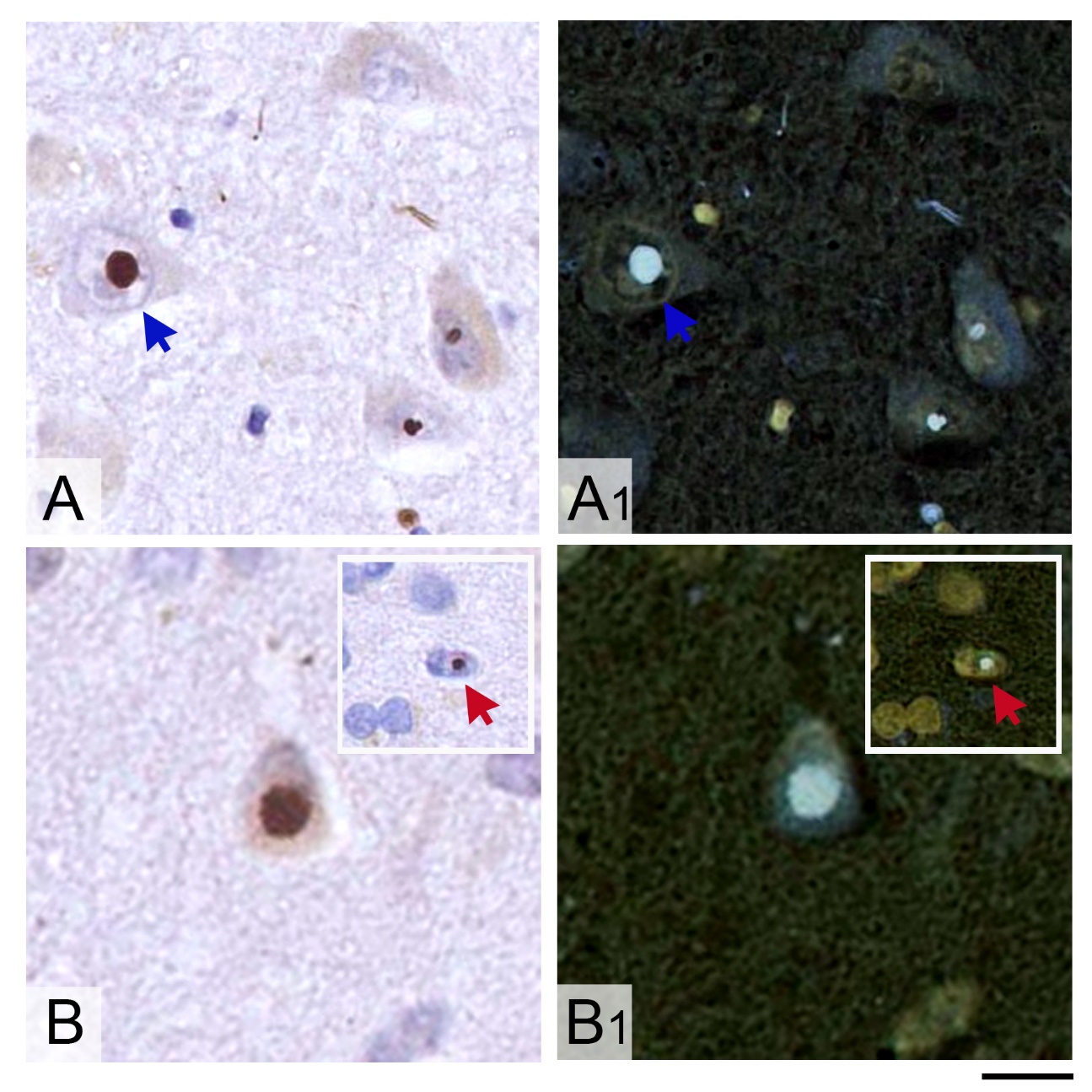
**

**Supplementary Figure 1. Cases with neuronal intranuclear inclusions and FTLD-FUS**

**
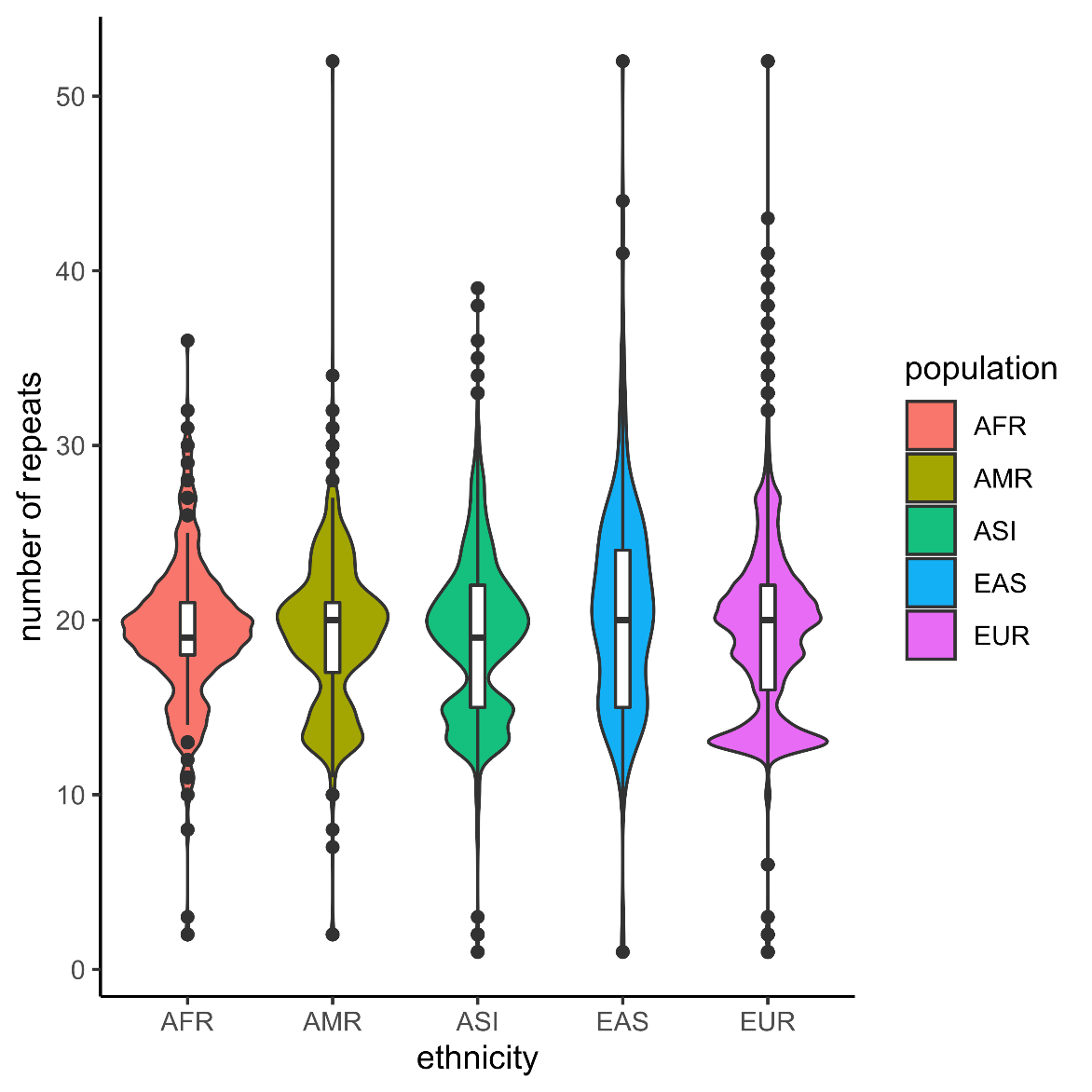
**

| **Ethnicity** | **n** | **Median number of repeats (IQR)** |
| --- | --- | --- |
| **European (EUR)** | 17126 | 20 (16 - 22) |
| **East Asian (EAS)** | 113 | 20 (15 - 24) |
| **American (AMR)** | 321 | 20 (17 - 21) |
| **South Asian (ASI)** | 2395 | 19 (15 - 22) |
| **African (AFR)** | 581 | 19 (18 - 21) |

**Supplementary Figure 2. Distribution of repeat expansion sizes across different ethnic groups within 100,000 Genomes Project.**


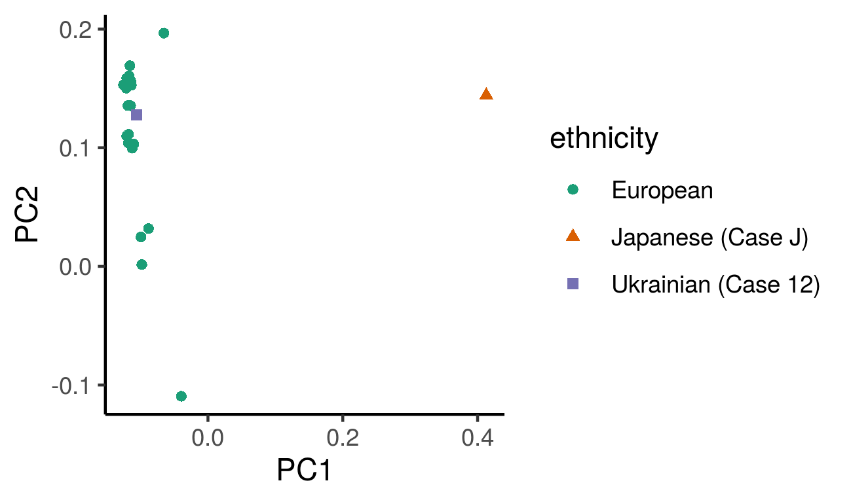

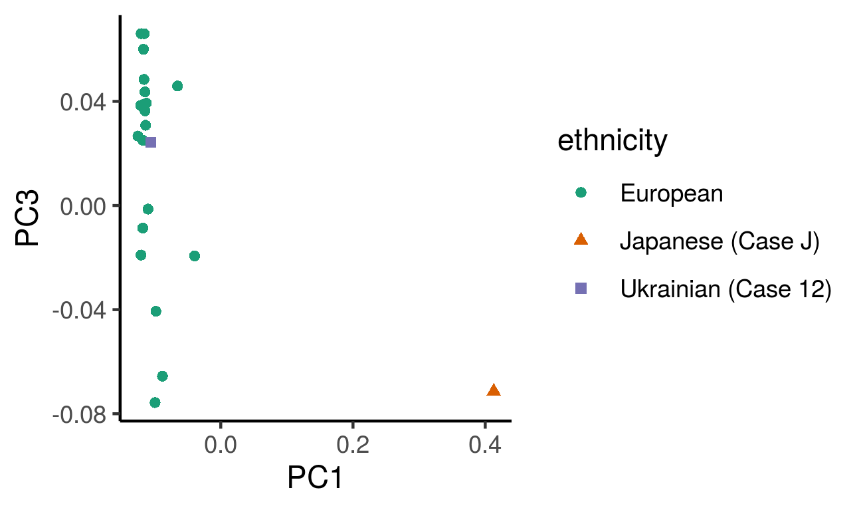

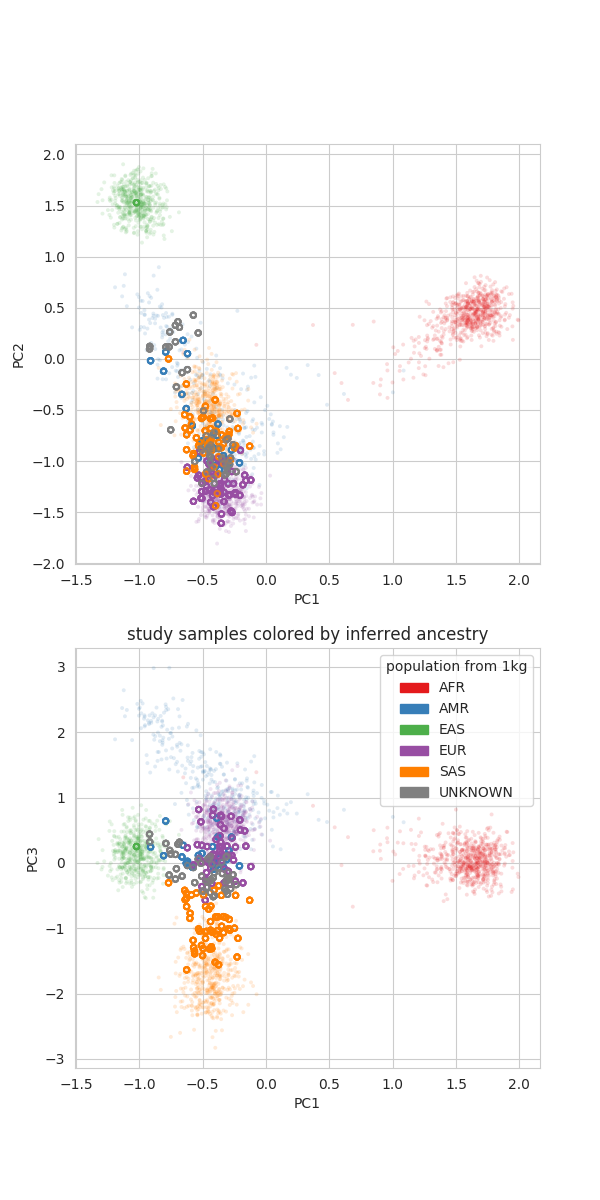


**B**

**A**

**Supplementary Figure 3. Principal component analysis stratified by self-reported ethnicity (A) and inferred ancestry compared to 1000 Genomes Project (1kg) (B).**

|  |  |  |  | **Pathologically-confirmed cases of NIID** | | | | | | | | | | | | | Cases with neuronal intranuclear inclusions pathologically | | | | | | | | |
| --- | --- | --- | --- | --- | --- | --- | --- | --- | --- | --- | --- | --- | --- | --- | --- | --- | --- | --- | --- | --- | --- | --- | --- | --- | --- |
| **POSITION GRCh38** | **SNP ID** | **REF** | **ALT** | **Case 12** | **Case J** | **Case 1** | **Case 2** | **Case 3** | **Case 4** | **Case 6** | **Case 7** | **Case 8** | **Case 9** | **Case 10** | **Case 11** | Case 2-1 | | Case 2-2 | Case 2-3 | Case 2-4 | Case 2-7 | Case 2-9 | Case 2-10 | Case 2-11 | Case 2-12 |
| 121164607 | rs6683357* | G | A | A | A | A | G | A | A | A | A | G/A | A | G/A | G | G/A | | G/A | G/A | G/A | G/A | A | G/A | A | G/A |
| 121184898 | rs4844381* | C | T | T | T | T | C | T | T | T | T | C/T | T | C/T | C | C/T | | C/T | C/T | C/T | C/T | T | C/T | T | C/T |
| 121230790 | rs6600662* | G | A | A | G/A | A | G | A | G/A | A | A | G/A | G/A | G/A | G | G/A | | G | G/A | G/A | G/A | G/A | G | A | G/A |
| 121280613 | rs11249433* | A | G | G | A/G | G | A/G | G | A/G | A/G | G | A/G | A/G | A/G | A | A/G | | A | A/G | A/G | A/G | G | A | A/G | A/G |
| 121349358 | rs1851250 | A | G | A | A | A | A/G | A | A | A | A | A | A | A | A | A | | A | A | A | A | A | A/G | A | A |
| 145383239 | rs12086156 | C | T | C | C/T | C | C | C | C | C | C | C | C | C | C | C | | C | C | C | C | C | C | C | C/T |
| 145394955 | rs10797649* | C | A | C/A | C | C | A | C | A | C/A | C/A | C/A | C/A | A | A | C | | C | A | C/A | A | C | C/A | C/A | C |
| 145395604 | rs12091564 | T | C | T | T/C | T | T | T | T | T | T | T | T | T | T | T | | T | T | T | T | T | T | T | T/C |
| 145428296 | rs6661602* | C | T | T | T | C | T | C/T | T | C/T | T | C/T | C/T | T | T | C/T | | C | T | T | T | C | T | T | T |
| 145444556 | rs4636400* | T | G | T/G | T/G | T | T | T | T/G | T/G | T/G | T/G | T | G | T | T | | T | G | T/G | G | T | T | T/G | T |
| 145479219 | rs833943 | C | T | T | T | T | T | T | T | C/T | T | T | T | T | T | T | | T | T | T | T | T | T | T | C/T |
| 145517063 | exm2265040 | G | A | A | A | A | A | A | A | G/A | A | A | A | A | A | A | | A | A | A | A | A | A | A | G/A |
| 146501348 | rs10900384* | C | T | C | C | C | C/T | T | C | C | T | C | T | C/T | C/T | T | | C/T | C/T | C | T | C/T | C | T | C |
| 146506691 | rs12142005* | T | C | T | T | T | T/C | C | T | T | C | T | T/C | T | T/C | C | | T/C | T | T | C | T | T | C | T |
| 146508774 | rs1853782* | C | T | T | T | C | T | T | C/T | T | T | C/T | T | T | T | T | | C/T | T | T | T | T | C/T | T | T |
| 146508934 | rs12122100* | C | T | C | C | C | C/T | T | C | C | T | C | C/T | C | C/T | T | | C/T | C | C | T | C | C | T | C |
| 146589758 | rs750467* | C | T | C | T | C/T | C | C | C | C/T | C/T | C | C/T | C | C | C | | C/T | C | C/T | C | C | C | C | C |
| 146626922 | rs6937* | T | C | T | C | T/C | T | T | T | T | T/C | T | T | T | T | T | | T/C | T | T/C | T | T | T | T | T/C |
| 146628635 | rs1837983* | A | G | A | G | A/G | A | A | A/G | A/G | A/G | A/G | A/G | A | A | A | | A/G | A/G | A/G | A | A | A | A | A |
| 146643555 | rs1348316* | A | G | A | G | A/G | A | A | A/G | A/G | A/G | A/G | A/G | A | A | A | | A/G | A/G | A/G | A | A | A | A | A |
| 146791729 | rs11239984* | G | A | A | G | G/A | G | G/A | G/A | G/A | G | G | G | G/A | G/A | G/A | | G | G/A | G/A | G | G | A | G | G/A |
| 146813984 | rs2883320* | T | G | T | T/G | T | G | T/G | T | T | G | T | T/G | T/G | T/G | T/G | | T/G | T | T/G | G | T/G | T | G | T |
| 146828670 | rs78674015 | C | T | C | C | C/T | C | C | C | C | C | C | C | C | C | C | | C | C | C | C | C | C | C | C |
| 146849143 | rs2883318* | A | C | A | A/C | A/C | A | A | A/C | A/C | A | A/C | A/C | A | A | A | | A/C | A/C | A | A | A | A | A | A/C |
| 146867044 | rs11240021* | C | T | T | C/T | C/T | T | T | C/T | C/T | T | C/T | C/T | T | T | T | | C/T | C/T | T | T | T | T | T | C/T |
| 146927395 | rs4950437* | A | C | A | C | A/C | C | A/C | A | A | C | A | C | C | A/C | C | | C | A | A | C | C | A | C | A/C |
| 146930254 | rs7525162* | A | G | A | A/G | A/G | A/G | A | A | A | A | A | A/G | A | A | A | | A/G | A | A | A | A | A | A | A/G |
| 146970946 | rs10900374* | G | A | A | G | G/A | G | G/A | G/A | A | G | G/A | G | G | G/A | G | | G | A | A | G | G | A | G | G/A |
| 146978395 | rs661678* | T | C | T | T/C | T | T/C | T/C | T | T | C | T/C | T/C | T/C | T/C | C | | T/C | T | T | C | C | T/C | C | T |
| 147192818 | rs4950476* | C | T | C/T | C/T | T | C/T | C/T | C | T | C/T | C | C | C/T | C/T | C | | C | T | T | C/T | C/T | T | T | T |
| 147194005 | rs11577892* | T | G | T/G | T/G | T | T | T | G | T | T/G | T/G | G | T/G | T/G | T/G | | G | T | T | T/G | T/G | T | T | T |
| 147205770 | rs6593803* | T | C | T/C | C | C | C | C | T | C | C | C | T | T/C | C | T/C | | T | C | C | T/C | T/C | C | C | C |
| 147220045 | rs12032789* | C | A | C/A | C | C | C | C | A | C | C | C | C/A | C/A | C | A | | A | C | C | C/A | C/A | C | C | C |
| 147321889 | rs76359988 | A | C | A | A | A | A | A | A | A | A | A/C | A | A | A | A | | A | A | A | A | A | A | A | A/C |
| 147322813 | rs12401663 | A | G | A | A | A | A | A | A | A | A | A/G | A | A | A | A | | A | A | A | A | A | A | A | A/G |
| 147323301 | rs7518323 | T | C | T | T | T/C | T | T | T | T/C | T/C | T/C | T | T | T | T | | T | T/C | T | T | T | T | T | T/C |

**Supplementary Table 1. Haplotype blocks within the *NOTCH2NL* region of interest**
